# Comparison of Oviposition and Adult Trapping to Monitor *w*Mel Introgression for *Wolbachia-*Based Vector Control

**DOI:** 10.4269/ajtmh.26-0050

**Published:** 2026-07-21

**Authors:** Elisabeth Nelson, Thiago N. Pereira, Erica Milena de Castro Ribeiro, Bianca Daoud Mafra e Silva, Carolina Camillo, Thiago Rodrigues da Costa, Mauro M. Teixeira, Albert I. Ko, Derek A. T. Cummings, Luciano A. Moreira

**Affiliations:** ^1^ Department of Epidemiology of Microbial Diseases, Yale School of Public Health, New Haven, Connecticut, USA;; ^2^ World Mosquito Program, Fundação Oswaldo Cruz Instituto Rene Rachou, Belo Horizonte, Brazil;; ^3^ World Mosquito Program, Instituto Oswaldo Cruz (Fiocruz), Rio de Janeiro, Brazil;; ^4^ Department of Biochemistry and Immunology, Federal University of Minas Gerais, Belo Horizonte, Brazil;; ^5^ Instituto Gonçalo Moniz, Fundação Oswaldo Cruz (Fiocruz), Salvador, Brazil;; ^6^ Emerging Pathogens Institute and Department of Biology, University of Florida, Gainesville, Florida, USA;; ^7^ Departments of Epidemiology and Biomedical Engineering, Johns Hopkins University, Baltimore, Maryland, USA

## Abstract

*Wolbachia* introgression into *Aedes aegypti* (*Ae. aegypti*) mosquito populations is an effective strategy for preventing dengue and is currently under evaluation for WHO prequalification. The standard protocol for trials and deployments currently being conducted in Brazil by the World Mosquito Program (WMP) for long-term monitoring of *Wolbachia* introgression involves the use of BG-Sentinel traps (BG-traps; Biogents AG [Regensburg, Germany]), which are labor-intensive and costly. More affordable alternatives, such as oviposition traps (ovitraps), have been used in other settings; however, a comparison of the WMP protocol for *Wolbachia* surveillance has not been performed. As part of the EVITA dengue trial, *Ae. aegypti* eggs and adults were collected from 124 ovitraps and 237 BG-traps across 12 clusters in Belo Horizonte, Brazil, between March and May 2023. Eggs collected from ovitraps were reared to L3 and L4 larvae, and *w*Mel *Wolbachia* was detected using quantitative polymerase chain reaction testing in larvae and adult mosquitoes from BG-traps. Mixed-effects models were used to compare cluster-level estimates of *w*Mel introgression derived from ovitrap and BG-trap data over time. Among 3,675 larvae reared from ovitraps, *w*Mel prevalence was 0.50 (95% CI: 0.48–0.51), whereas 1,244 adults from BG-traps had a prevalence of 0.45 (95% CI: 0.42–0.48). Cluster-level introgression estimates from larvae and adults were strongly correlated (Spearman’s *r* = 0.70; *P* <0.001). Multivariate models incorporating current- and previous-month ovitrap data and mosquito abundance revealed strong agreement between ovitrap-based predictions and BG-trap estimates (*r*_s_ ≥0.98). These findings reveal that ovitrap-based surveillance provides a low-resource, efficient, and precise alternative for monitoring *Wolbachia* introgression as interventions are scaled in high-burden dengue settings.

## INTRODUCTION

*Wolbachia* introgression into *Aedes aegypti* (*Ae. aegypti*) mosquito populations has been shown to be highly effective in preventing dengue. In the first cluster-randomized controlled trial of this intervention conducted in Yogyakarta, Indonesia, releases of *Wolbachia*-infected (*w*Mel strain) *Ae. aegypti* reduced virologically confirmed dengue by 77.1%, despite widespread contamination between intervention and control clusters.^1^ In large-scale releases in Rio de Janeiro, Brazil, an increasing reduction in dengue incidence was associated with increasing *Wolbachia* introgression.^2^ Reductions in dengue incidence of 71% were observed in areas where >60% *Wolbachia* introgression had occurred. Yet, a 20% decrease in dengue risk was also observed in areas with low *Wolbachia* introgression (10%–20%). Although evidence is accruing that reveals the effectiveness of *Wolbachia*-based interventions, scaling these interventions for widespread roll-out in low-resource settings will be a key challenge in the future.

Monitoring *Wolbachia* introgression during and after release of *w*Mel-infected *Ae. aegypti* is a key implementation barrier. Surveillance of *Ae. aegypti* populations is used to guide releases to achieve introgression. Modeling studies have suggested that once introgression levels are greater than 60%, the local spread of *Wolbachia* is highly likely to increase because of the frequency-dependent fitness advantage of *Wolbachia*-infected females.^3^ Studies have also suggested that *Wolbachia* introgression levels greater than 60% ensure a greater potential of achieving self-sustaining *Wolbachia* populations.^1^^,^^3^^–^^6^ Therefore, it is crucial to accurately estimate *Wolbachia* introgression to ensure successful, self-sustaining introgression into wildtype populations and successful prevention of arboviral transmission. Current interventions use an extensive network of mosquito traps to continuously monitor local mosquito populations. BG-Sentinel traps (BG-traps; Biogents AG [Regensburg, Germany]) are widely used for monitoring *Wolbachia-*based interventions; however, they are expensive and require a constant source of power, which in turn restricts where they can be deployed.^7^^,^^8^

Oviposition traps (ovitraps) represent an alternative monitoring method to help overcome the cost and resource barriers to scale-up. However, there is limited information on the accuracy of ovitraps in predicting *w*Mel *Wolbachia* introgression. de Jesus et al. conducted a study during ongoing releases in Rio de Janeiro to compare the efficacy of ovitraps with that of BG-traps.^7^ They found a low (<0.1%) sampling error using ovitraps in a semi-field enclosure and comparable introgression predictive abilities between ovitraps and BG-traps in field-based experiments. Ovitraps have been used for Wolbachia monitoring in several interventions, including the first wMel release in Australia and large-scale releases of wAlbB in Malaysia, with some programs relying exclusively on ovitrap data.^9^^,^^10^ Despite these results, the comparative predictive ability of ovitraps at estimating *w*Mel *Wolbachia* introgression has yet to be evaluated at larger spatial scales and using routine surveillance networks.

The authors of the present study had the opportunity to address the question of the ovitrap’s predictive ability in a large field setting, using existing *Ae. aegypti* surveillance networks during the implementation of the A Cluster-randomized Trial to EValuate the Efficacy of Wolbachia-InfecTed Aedes aegypti Mosquitoes in Reducing the Incidence of Arboviral Infection in Brazil (EVITA) dengue trial, a cluster-randomized controlled trial aimed at evaluating the effectiveness of releasing wMel-infected *Ae. aegypti* in preventing arboviral infection in the city of Belo Horizonte, Brazil.^11^ As part of this trial, BG-trapping was performed in intervention and control clusters, in parallel to monitoring ovitraps, which were performed by local public health authorities. The aim for the present study was to correlate *w*Mel prevalence estimates measured using ovitraps with those measured using BG-traps and evaluate the accuracy of ovitrap-based estimates of introgression in predicting BG-trap estimates.

## MATERIALS AND METHODS

### EVITA dengue trial.

**Figure 1.**
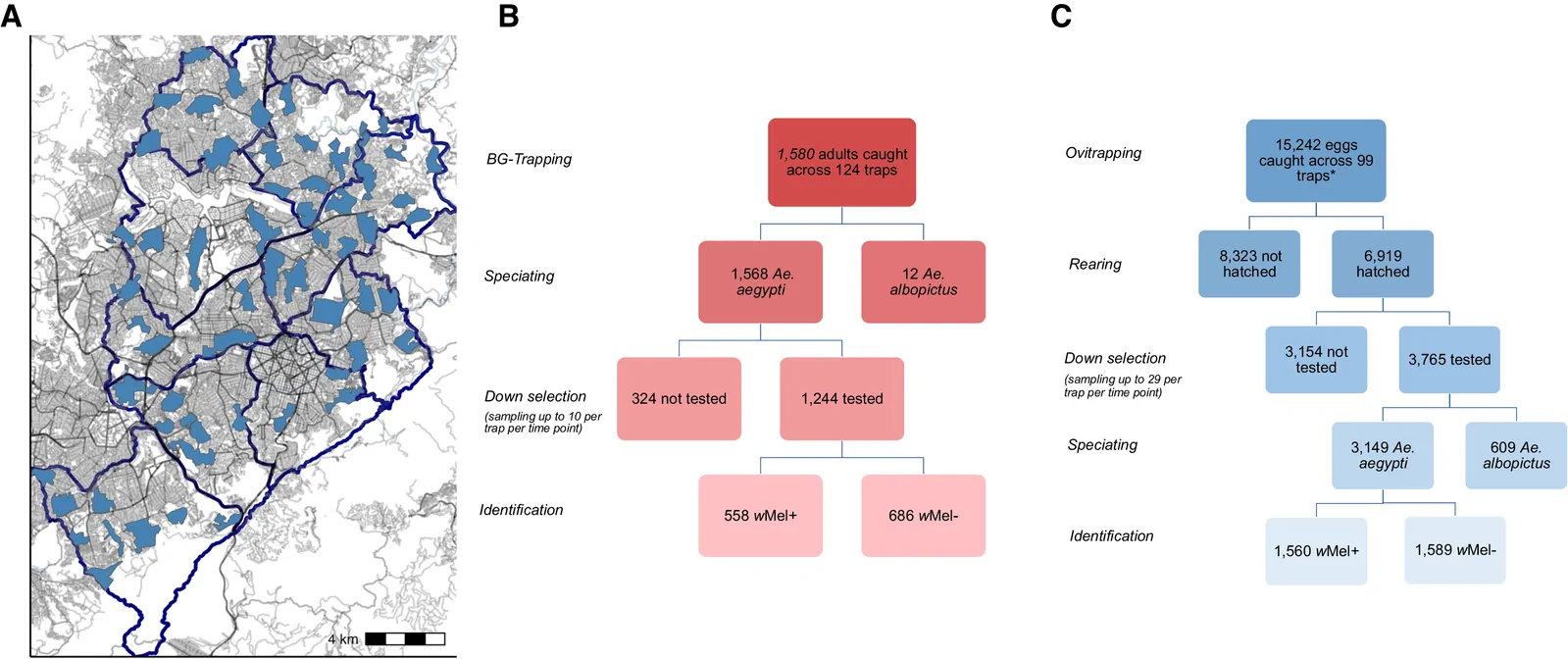
Experimental study design. (**A**) EVITA trial clusters in Belo Horizonte, Brazil, with road density and size indicated by grayscale.^11^ The map was produced in R (R Foundation; Vienna, Austria) using a base layer from Belo Horizonte Geoprocessamento’s catalog of metadata for the Belo Horizonte municipality and maps of rivers and small, medium, and large streets from the OpenStreetMap package in R (R Foundation).^14^^,^^15^ (**B**) *Aedes* adult mosquito testing protocol from BG-Sentinel trap (BG-trap; Biogents AG [Regensburg, Germany]) collection. (**C**) *Aedes* larvae mosquito testing protocol from oviposition trap (ovitrap) collection. Down selection refers to the sampling of trap collections for testing: up to 10 *Aedes aegypti* adults per BG-trap and up to 29 *Aedes* larvae per ovitrap. Eggs were counted by the Municipal Ministry of Health in March and April and by the first author in May.

The present study was conducted in Belo Horizonte, the sixth most populous city in Brazil, located in the Southeastern state of Minas Gerais.^12^ Located at 850 m above sea level, the climate is primarily tropical, receiving ∼50 inches of rainfall per year, with an arid winter season.^13^ This city was selected as the site for the EVITA dengue trial, a cluster-randomized control trial aimed at evaluating the effectiveness of releasing *w*Mel *Ae. aegypti* adults on the risk of serologically ascertained arboviral infection among schoolchildren that is scheduled to end in the late spring of 2026.^11^ Within the city, 58 school-based clusters were identified, of which 29 were randomly assigned to an intervention arm and 29 were randomly assigned to a control arm (Figure 1A). Clusters ranged from 0.44 to 2 km^2^ in size and were separated by at least 200 m or a natural barrier from another cluster to mitigate the risk of contamination of control clusters with *w*Mel *Ae. aegypti* from intervention clusters.

The nine major municipal regions of the city were geographically grouped into three phases of staggered releases to improve operational feasibility: Phase 1 underwent releases between January 2021 and January 2022 in 10 clusters, Phase 2 underwent releases from April 2021 to April 2022 in nine clusters, and Phase 3 underwent releases between May 2021 and May 2022 in 10 clusters. Intervention and control clusters were balanced within each phase. Additional information on the trial can be found in the Collins et al. trial protocol.^11^

### Study sites.

The nine intervention clusters, one from each of the city’s main areas (three from each of the trial release phases), and three control clusters, one from each of the trial release phases, were selected by an unblinded member of the World Mosquito Program (WMP) team and assigned a letter to ensure continued blinding. Samples were collected from March to May 2023 (epidemiological weeks 10, 14, and 18) during the monitoring phase of the EVITA trial. Sample processing then occurred between mid-May and late July 2023. Because *w*Mel-positive eggs do not remain viable when desiccated as long as wild-type (*w*Mel-negative) eggs, it was determined that March was the earliest time at which collections could begin to be stored before May processing.^16^ To maintain blinding for the trial, no spatial information on the traps other than their cluster was collected.

### BG-Sentinel trapping.

The BG-trap, which is currently the gold standard for capturing *Ae. aegypti* mosquitoes, uses visual attractants to the human body to capture host-seeking females.^7^^,^^17^^,^^18^ As part of the EVITA trial, BG-Sentinel Traps version 2 (with flaps) were placed in a gridded arrangement in all clusters, with up to 16 traps per km^2^.^11^ Traps were preferentially placed in commercial locations, such as stores, within 1 meter of the ground. The traps used in the trial do not contain any chemical bait or carbon dioxide; instead, they attract females solely through their dark color, which has been validated by WMP and used as a standard procedure in their interventions globally.^19^ Given that the present study took place during the monitoring phase of the EVITA trial, traps operated for 15 days per month, and samples were collected after this 15-day window, in accordance with standard WMP protocols.^5^ Trap collections were processed at the WMP Biofactory in Belo Horizonte, Brazil, where all mosquitoes were morphologically identified at the species level and recorded. Up to 10 *Ae. aegypti* samples per trap (determined by the WMP for operational feasibility) were then sent to the WMP Diagnostic Laboratory at the Instituto René Rachou (Fiocruz; Rio de Janeiro, Brazil) for *w*Mel identification.^20^

### Oviposition trapping.

Ovitraps consist of a wooden paddle, ∼15 cm × 2.5 cm, submerged in a container of hay-infused water.^21^^,^^22^ Belo Horizonte’s municipal government has maintained a surveillance network of ovitraps, placed at intervals of 400 m (approximately nine traps per km^2^), which has been monitored monthly for arboviruses and mosquito abundance since 2006. The traps were installed outside of buildings. Staff of the Municipal Ministry of Health check the traps biweekly, with traps installed for 7 days at a time every 2 weeks in an alternating pattern around the city. There were 5 to 15 ovitraps per cluster, which have been found to collect, on average, 22 to 42 eggs (all mosquito species) per trapping cycle.

The Ministry of Health delivered ovitrap paddles from the study clusters to the WMP Biofactory, where they were sorted by cluster and time point. Eggs were hatched in a controlled environment at 28°C ± 2°C, 82% ± 2% relative humidity, and a 12-hour light/dark regimen. Larvae were fed a liquid solution of liver and yeast paste. L3 and L4 stage larvae samples were collected in tubes with a solution of 80% ethanol and taken to WMP Diagnostic Laboratory for *w*Mel identification.

The minimum sample size was determined to be ∼22 *Ae. aegypti* mosquitoes per ovitrap per time point to measure *Wolbachia* prevalence with 80% power. This was calculated using a two-sided power calculation for a proportion test (one sample) in the “pwr” package in R (R Foundation, Vienna, Austria), assuming that *w*Mel prevalence was at least 60% in all intervention clusters, 80% power, and 5% error (Supplemental Figure 1).^23^ Based on speciating ovitrap collections in the area, for a subset of ovitraps, ∼10% of collected eggs were *Aedes albopictus* (*Ae. albopictus*)*.* To conservatively account for this, 28 to 29 larvae were randomly collected from each ovitrap sample to ensure 22 *Ae. aegypti* samples. If 28 to 29 larvae were not available for a collection, the total number of larvae available was used.

To understand the effect of egg storage time on *w*Mel DNA degradation and quantitative polymerase chain reaction (qPCR) detection, the density and DNA extraction failure rate were measured in *w*Mel eggs stored for 1, 2, 4, 6, 8, or 16 weeks. Eggs from the WMP’s *w*Mel *Wolbachia*-positive *Ae. aegypti* colonies were used for testing. The WMP maintains several colonies for experiments and quality assurance, with eggs collected and aggregated by colony type weekly for storage. All eggs were stored in a controlled environment at the WMP’s laboratories in Instituto René Rachou (Fiocruz) in Belo Horizonte at 28°C ± 2°C, 82% ± 2% relative humidity, and a 12-hour light/dark regimen. Eggs were collected on filter paper from oviposition cups and dried for a period of at least 2 days. The experiment was conducted three times, with 74 eggs of each age per replicate, using 384-well qPCR plates. The WMP’s *w*Mel *Wolbachia* detection assay protocol was used for larvae and adult *Aedes* mosquitoes, as detailed below in the *Estimation of wMel Prevalence* section. Immediately before pipetting the samples from the DNA extraction plates into the qPCR wells with two 2-mm glass/silica beads and 25 *µ*L of Squash Buffer/Proteinase K solution, the DNA extraction plates were centrifuged at 4,000 rpm for 5 minutes. This was a vital step to ensure that egg DNA was pipetted into the qPCR plates. An individual mosquito was scored as positive for ribosomal protein S17 (RPS) if amplification occurred and the qPCR cycle threshold (Ct) value was less than 35, and as positive for Wolbacia surface protein (WSP) if amplification occurred and the Ct value was less than 30.

### Estimation of *w*Mel prevalence.

All samples were processed using the WMP’s standardized protocols, which have been validated throughout its international study sites.^20^ For DNA extraction, adults and larvae were individually macerated and homogenized in Squash Buffer Mix (pH 8.2; 10 mM Tris hydrochloride; 25 mM sodium chloride; 1 mM ethylenediaminetetraacetic acid) supplemented with proteinase K (200 *µ*g/mL), followed by incubation at 56°C for 5 minutes to activate the enzyme. Extraction was interrupted by enzyme inactivation at 98°C for 5 minutes. The plate was then cooled to 12°C.

For *w*Mel identification, adults and larvae were screened using the Promega qPCR kit (Promega Corporation; Madison, WI, USA) on a QuantStudio12k (Thermo Fisher Scientific; Waltham, MA, USA). The qPCR cycling program consisted of a preincubation at 95°C for 5 minutes, followed by 40 cycles of polymerase chain reaction amplification (95°C for 3 seconds and 60°C for 30 seconds), and a cooling-down step at 37°C for 30 seconds. *Ae. aegypti* and *w*Mel were identified using the endogenous control housekeeping gene *RPS17* for *Ae. aegypti* and the gene encoding *Wolbachia* surface protein *WSPTM2* using the following primers and probes: *RPS17* Forward: 5′-TCC GTG GTA TCT CCA TCA AGC T-3′, Reverse: 5′-CAC TTC CGG CAC GTA GTT GTC-3′ and Probe 5′-HEX/CAGGAGGAG/ZEN/GAACGTGAGCGCAG/3lABkFQ-3′; *WSPTM2* Forward: 5′-CATTGGTGTTGGTGTTGGTG-3′ and Reverse: 5′- ACACCAGCTTTTACTTGACCAG-3′ and *WSPTM2* Probe 5′-FAM/TCCTTTGGA/ZEN/ACCCGCTGTG AATGA/3lAbRQSp-3′. Individual mosquitoes were determined to be positive for *w*Mel if the *WSP* qPCR Ct value was less than 29. They were identified as *Ae. aegypti* if the *RPS* qPCR Ct value was less than 28. Alternatively, *Ae. albopictus* were identified if the *RPS* qPCR Ct value was greater than 28; these mosquitoes were not included in *w*Mel prevalence calculations.

The individual trap-level prevalence of *w*Mel-positive *Ae. aegypti* out of the total number of *Ae. aegypti* tested was averaged by cluster and time point to calculate cluster-level introgression estimates, which is the scale at which the WMP monitors introgression to determine if additional releases are required. Data from ovitrap paddles that did not result in any hatched larvae (*N* = 178) were excluded because this may have been due to the paddles not being collected by the municipality at that time point or eggs falling off during transport.

## STATISTICAL ANALYSES

Statistical analyses were performed using R version 4.2.2 (R Foundation).^24^ An initial analysis to determine if a significant difference existed between the introgression estimates of each trap type was performed using a Wilcoxon signed-rank test, due to the nonparametric nature of the data, paired by cluster and time point. The correlation between the trap-type estimates was then tested using a Spearman-rank correlation test, which is used to measure the strength and direction of association between ranked variables and is robust to nonlinearity. To ensure that the spatial locations of the traps (which the authors did not have access to) did not affect the correlation observed, a sensitivity analysis was performed using bootstrap resampling of the BG-trap- and ovitrap trap-level *w*Mel prevalence estimates, randomly sampling three, six, nine, and 12 traps per cluster to calculate a cluster-level prevalence estimate at each time point. A Spearman-rank correlation test was then performed. This analysis was repeated 10,000 times, with replacement when fewer than the designated number of traps were present in the cluster, to replicate the statistical uncertainty associated with the inclusion of specific sets of spatial locations being sampled. The cluster-level prevalence estimates were plotted by time point of BG-traps (*x*-axis) and ovitraps (*y*-axis). The sensitivity and specificity of the ovitrap-estimated prevalences were then determined by cluster and time point, classifying prevalence estimates as meeting an introgression threshold of 60%, with BG-trap-based prevalence serving as the gold standard. A threshold of 50% was also investigated. Additionally, the within-cluster variance in estimated *w*Mel prevalences was visually examined by trap type and time point, using the ggplot2 package in R (R Foundation) to construct forest plots.^25^

To evaluate predictions of BG-trap results using ovitrap measurements, negative binomial generalized linear mixed-effects regression models were constructed, with the outcome variable of BG-trap estimated with *w*Mel introgression. Potential predictor and covariate variables included the ovitrap-estimated *w*Mel count, the ovitrap-estimated *w*Mel proportion, lags of the ovitrap count and proportion, the month, an interaction between the ovitrap proportion and month, and the cluster. The outcome variable of BG-trap-estimated *w*Mel introgression and the covariate of the ovitrap-estimated *w*Mel count were calculated by multiplying the proportion of *w*Mel-positive *Ae. aegypti* of the total number of *Ae. aegypti* tested with the total number of *Ae. aegypti* caught by the trap. This provided an estimate of the total number of *w*Mel captured by the trap and weighed the results of traps with more *Ae. aegypti* caught more heavily because these would provide more certainty. Given that ovitrapping targets a part of the mosquito life cycle that is shifted relative to the adult phase (either forward or backward in time), correlations among multiple count lags for each trap type were assessed using a Spearman-rank correlation test. Given the constrained time scale of the data (three monthly observations per trap type per cluster), only lags of ±1 and zero months were used. The variable with the highest correlation was then used as the primary predictor in the models.

All models were fitted with an offset equal to the log of the total number of *Aedes* caught in BG-traps in that cluster at that time point. An offset was used because it effectively rendered the outcome as a rate and allowed the authors to include uncertainty around estimates in traps with fewer total mosquitoes caught. All combinations of covariates over space and time were tested using cluster-level data to maintain blinding for the EVITA dengue trial. Models were constructed using the *glmer.nb* function in the *MASS* package in R (R Foundation).^26^ Model building and selection took place in two phases: 1) examining the relationship between BG-trap and ovitrap data by month, cluster, and covariate space; 2) using the best-fitting model for prediction to determine if ovitrap data are a good proxy for BG-trap data. Model selection in the second phase was performed using the Bayesian Information Criterion score (BIC) using all possible combinations of covariates. The predicted *w*Mel counts and introgression estimates from the best-fitting model were compared with the real BG-trap counts and introgression estimates using a Spearman-rank correlation test and sensitivity and specificity tests of the predicted *w*Mel counts or introgression estimates. Finally, the magnitude and directionality of the model’s error were investigated by comparing predicted *w*Mel counts with the real BG-trap counts. The error was calculated by subtracting the BG-trap count from the model-predicted count and dividing the result by the BG-trap count.

## RESULTS

### *w*Mel prevalence in BG-traps and ovitraps.

**Figure 2.**
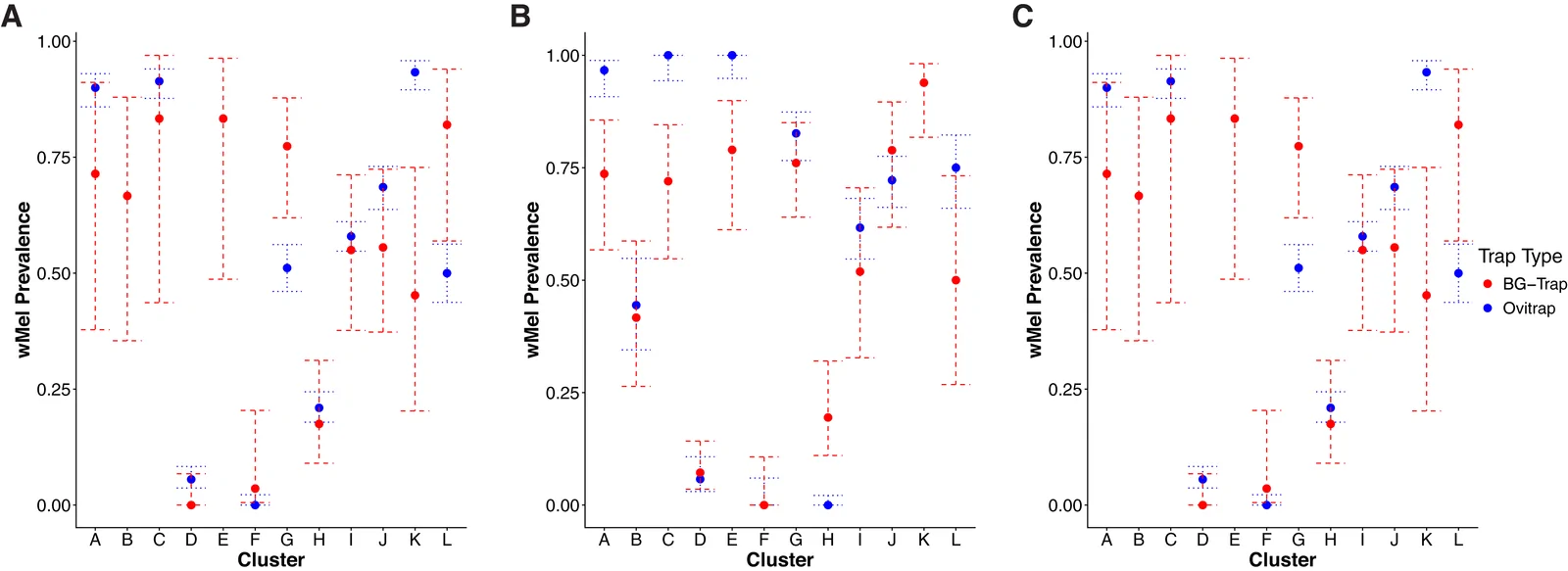
*w*Mel prevalence estimates by cluster and trap-type, March to May 2023. *w*Mel prevalence estimates in adult *Aedes aegypti* mosquitoes from BG-Sentinel traps (Biogents AG; Regensburg, Germany) and oviposition traps in (**A**) March, (**B**) April, and (**C**) May. The point estimate is surrounded by its 95% binomial CI. The cluster-level prevalence estimates represent averages of the trap-level proportions within that cluster at that time point.

During epidemiological weeks 10, 14, and 18 in March, April, and May 2023, 1,580 *Ae. aegypti* and *Ae. albopictus* adults were captured using BG-traps in the 12 selected clusters in Belo Horizonte (Figure 1B and Supplemental Table 1). Of these, 1,244 adult *Ae. aegypti* were tested, yielding an overall *w*Mel prevalence of 0.45 (0.42–0.48; cluster-level proportions are shown in Figure 2A–C and Supplemental Table 3; trap-level data are shown in Supplemental Figure 2). At these same time points, 6,919 larvae hatched and were reared from ovitraps, with an overall hatch rate of 45% (6,919/15,242; Figure 1C and Supplemental Table 2). The hatch rates were lower than expected, which are typically ∼80%; the highest rate was observed in March (52%), followed by similar rates in April (42%) and May (46%).^27^^–^^29^ These lower-than-expected hatch rates were likely due to the handling and storage time of the ovitrap paddles and, potentially, the increasingly cold temperatures with the onset of winter.^30^^,^^31^ A total of 3,675 larvae were tested, of which 3,149 were *Ae. aegypti* (86%) and 609 were *Ae. albopictus* (17%), with an overall *w*Mel prevalence of 0.50 (0.48–0.51) among *Ae. aegypti* tested (Figure 2A–C; Supplemental Figure 2 and Supplemental Table 4). *w*Mel prevalence estimates appeared to follow a similar pattern across clusters and time points between the two trap types (Figure 2A–C). After testing the effect of varying lengths of egg storage time, DNA extraction failure only increased from 2.7% after 1 week of storage to 9.96% after 8 weeks of storage and 10% after 16 weeks of storage (Supplemental Figure 3A). Eggs from ovitraps were stored up to 12 weeks at most before hatching. Additionally, a large decrease in the density of *w*Mel, relative to an *Aedes*-specific housekeeping gene, was not observed in samples stored between these time points (Supplemental Figure 3B).

### Correlation between *w*Mel prevalences in BG-traps and ovitraps.

**Figure 3.**
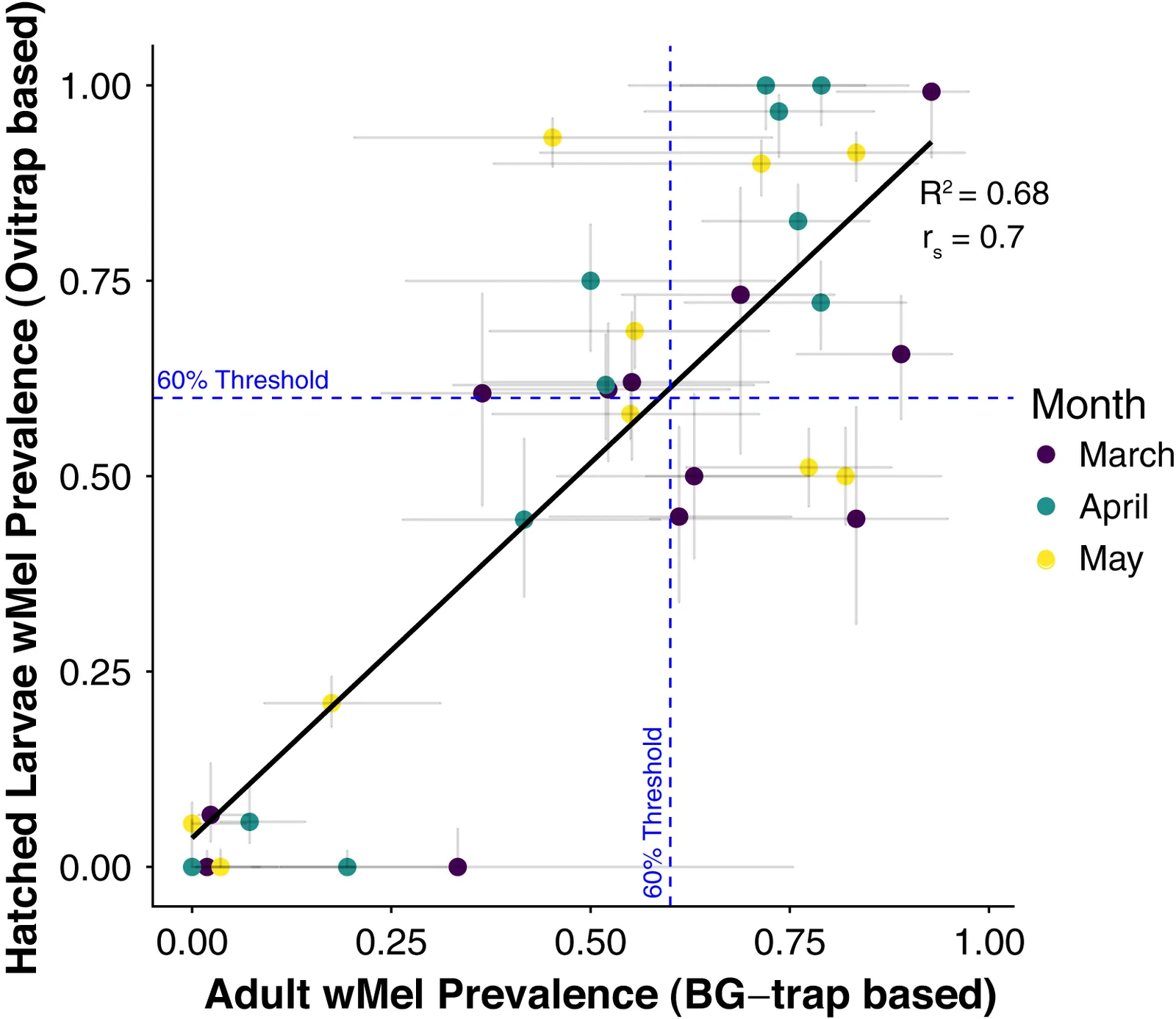
Correlation of cluster-level *w*Mel prevalence estimates in *Aedes aegypti* (*Ae. aegypti*) adults and larvae. The correlation between *w*Mel prevalence estimates from *Ae. aegypti* adults collected in BG-Sentinel trap (BG-trap; Biogents AG [Regensburg, Germany]) and larvae hatched from oviposition trap (ovitrap) collections by cluster and time point. The color of the point estimate indicates the time point: purple for March, blue for April, and yellow for May. In the *x*-direction, 95% binomial CIs from the BG-trap estimate extend from the point, whereas in the *y*-direction, 95% binomial CIs from the ovitrap estimate extend from the point. The black line represents the linear relationship between the BG-trap adult prevalence estimates and the ovitrap hatched larvae prevalence estimates, calculated using a linear model, with the corresponding linear slope, adjusted *R*^2^, and Spearman-rank correlation. The blue dotted lines indicate a 0.6 (60%) *w*Mel introgression threshold.

No significant differences were found between the ovitrap and BG-trap cluster-level *w*Mel prevalence estimates at each time point (V = 265; *P* = 0.16) using a Wilcoxon signed-rank test. Spearman-rank correlation tests revealed a high correlation between ovitrap and BG-trap cluster-level estimates of *w*Mel prevalence at each time point (r_s_ = 0.70; *P* = 6.71e-06; *R*^2^ = 0.68), indicating a strong rank correlation (Figure 3). Using bootstrap resampling of the ovitrap- and BG-trap-level data sampling from three to 12 traps of each type per cluster to estimate cluster-level prevalences (Supplemental Figure 4), a similar correlation was found between ovitrap and BG-trap cluster-level estimates of *w*Mel prevalence over time (r_s_ = 0.61–0.67). Ovitraps correctly classified the introgression threshold in 64% of estimates. However, 12 estimates (36%) were misclassified, with seven overestimated and five underestimated by ovitraps. The overall sensitivity of ovitrap prevalence classification was 59%, and the specificity was 69%, across clusters and time points. The sensitivity and specificity appeared to be lowest in March, the month with the greatest number of misclassifications (Supplemental Table 5). When the introgression threshold was lowered to 50%, only four misclassifications occurred. When a further reduced threshold of 36% was used, there was no discordance between the two trap types’ estimates.

Examining the within-cluster variation of prevalence estimates (i.e., the variance in the trap-level estimates), similar within-cluster variation was observed between ovitraps (0.13) and BG-traps (0.12) across clusters and time points. When viewed by time point, ovitraps had slightly lower within-cluster variance in April and May (0.17 in March; 0.09 in April; 0.11 in May) than BG-traps (0.11 in March; 0.13 in April; 0.13 in May); however, the same finding was not observed in March (Supplemental Figure 5).

### Factors associated with BG-trap-based *w*Mel introgression.

To determine the best-fitting model of the *w*Mel-positive *Ae. aegypti* captured using BG-traps (outcome) and *w*Mel-positive *Ae. aegypti* captured using ovitraps (predictor), the correlation by cluster of ±1-month temporal lags between ovitrap and BG-trap *w*Mel-positive mosquito counts was tested using a Spearman-rank correlation test (Supplemental Table 6). A slightly positive correlation was observed between BG-trap and ovitrap counts averaged across locations over time (*r_s_* = 0.12). A strongly positive correlation was observed between BG-trap counts and a –1-month lagged (previous collection time point) ovitrap count (*r_s_* = 0.78), as well as between a +1-month lagged BG-trap count (next collection time point) and ovitrap count (*r_s_* = 0.78). The *Ae. aegypti* life cycle takes ∼7 to 10 days to complete, and the adult lifespan is ∼3 weeks; therefore, eggs from one month would likely be adults the next, and vice versa. Therefore, it is both statistically and biologically plausible that there is a lagged temporal correlation between BG-trap and ovitrap *w*Mel-positive counts. Given that the study model was designed to investigate the association with the current month’s BG-trap *w*Mel count, a –1-month lagged ovitrap *w*Mel count was used as the primary predictor variable in all models.

Using a negative binomial regression approach, the relationship between BG-trap and ovitrap data was examined across months, clusters, and covariate space. Across models, the average coefficient for the –1-month lagged ovitrap *w*Mel count was –0.00057. Given that the outcome of the model was a proportion (introgression), an incremental increase in the number of *w*Mel-positive larvae in an ovitrap would likely not affect the overall prevalence of *w*Mel in the cluster; therefore, a coefficient of almost zero is reasonable. The prevalence of *w*Mel in ovitraps had an average coefficient of 1.178 across models, showing a significant association with increases in the outcome proportion in BG-traps. The –1-month-lagged ovitrap proportion exhibited greater variability across models, with an average coefficient of 1.354. Therefore, it appears that the prevalence of *w*Mel in ovitraps in both the current month and the previous month was highly associated with the current month’s prevalence of *w*Mel in BG-traps, when accounting for the previous month’s ovitrap *w*Mel count. Finally, including an interaction between ovitrap prevalence and month did not appear to greatly improve the fit of the model. Although surprising, this finding reinforces the stability of the association between ovitrap prevalence and BG-trap prevalence through time.

### Predictive model of BG-trap-based *w*Mel introgression using ovitrap-based inputs.

**Figure 4.**
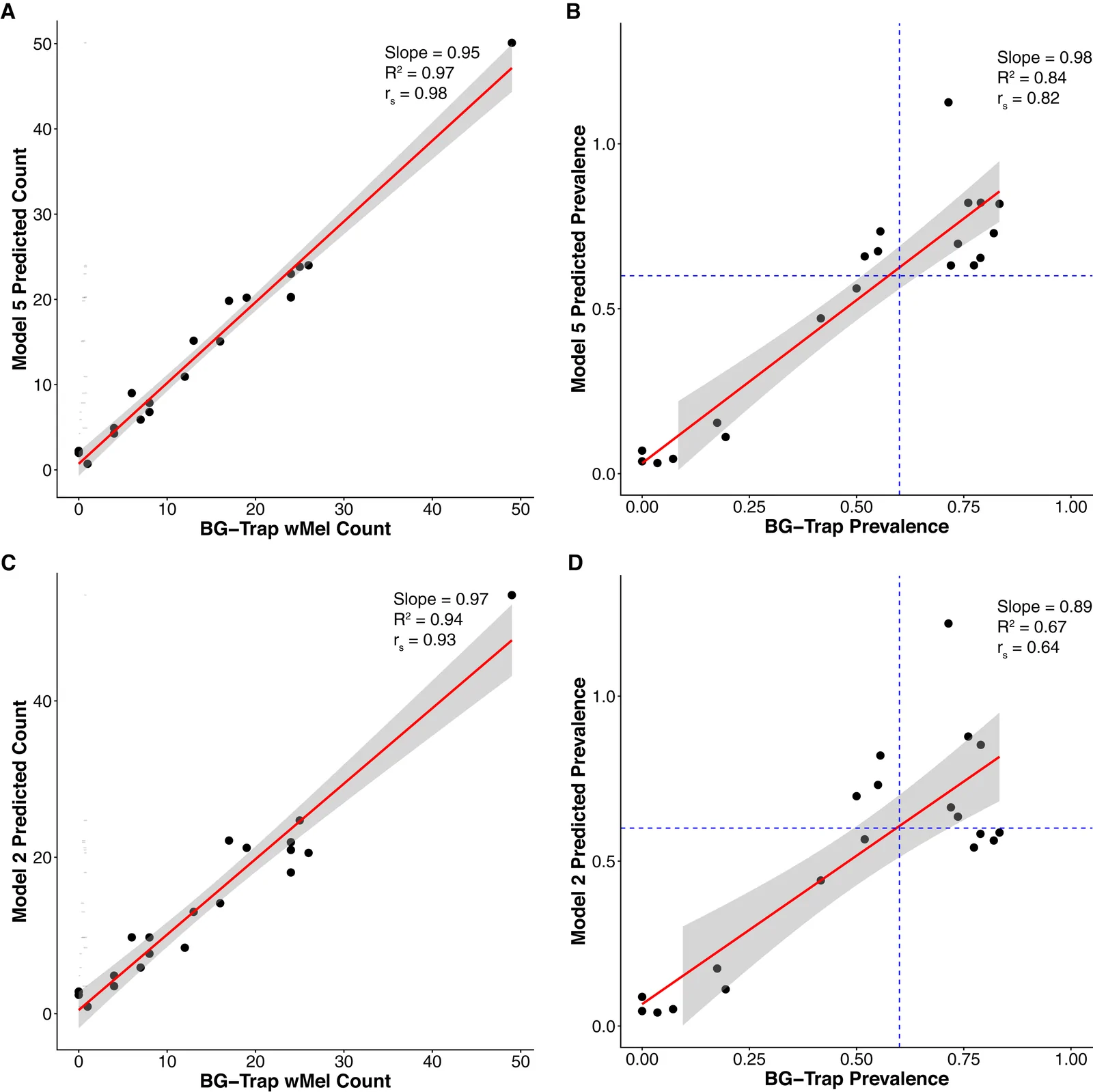
Model prediction. Correlation of (**A**) BG-Sentinel trap- (BG-trap; Biogents AG [Regensburg, Germany]) and Model 5-predicted *w*Mel counts and (**B**) introgression estimates by cluster and time point. Correlation of BG-trap- and Model 2-predicted (**C**) *w*Mel counts and (**D**) introgression estimates by cluster and time point. The red lines represent the linear relationship between the BG-trap- and model-predicted collections and estimates, calculated using a linear model, with corresponding slope, adjusted *R*^2^, and Spearman-rank correlation.

The best-fitting model, as determined by BIC scores, included the covariates of –1-month-lagged ovitrap *w*Mel count, ovitrap *w*Mel prevalence, –1-month-lagged ovitrap *w*Mel prevalence, and an offset for the total number of *Ae. aegypti* caught using BG-traps (Model 5 in Table 1, bolded). Additionally, the model that included only the –1-month-lagged ovitrap *w*Mel count, ovitrap *w*Mel prevalence, and an offset for the total number of *Ae. aegypti* caught using BG-traps (Model 2 in Table 1, bolded) fit almost as well and may provide a simpler model for prediction by requiring slightly less information. Using these models, prediction accuracy was compared with the gold-standard approach of BG-traps in estimating *w*Mel counts and introgression. As shown in Figure 4, the predicted *w*Mel counts were highly positively correlated with the true counts from BG-traps for both models (Model 5: *r_s_* = 0.98, *P* = 1.53e-14; Model 2: *r_s_* = 0.93, *P* = 4.15e-09). The model-predicted introgression estimates were also highly positively correlated with the BG-trap introgression estimates (Model 5: *r_s_* = 0.82, *P* = 0.11e-05; Model 2: *r_s_* = 0.64, *P* = 0.0024); however, Model 2 was slightly less correlated than the raw ovitrap introgression estimates (*r_s_* = 0.70). Overall, the model predicted the real *w*Mel counts very well, with an error of 0.0004 for Model 5 and 0.004 for Model 2 across clusters and time points (Supplemental Table 7). Both models yielded slightly underestimated counts in April (Model 5 error = –0.02; Model 2 error = –0.002) and slightly overestimated counts in May (Model 5 error = 0.02; Model 2 error = 0.01).

**Table 1:** Regression model coefficients of a model predicting *w*Mel *Wolbachia* prevalence, as measured using BG-Sentinel traps

Model	1	2	3	4	5	6	7	8	9
Fixed effects									
Lag 1 ovitrap *w*Mel count	3.16E-05	**4E-03**	−1.9E-03	−3.6E-04	**4.9E-04**	5E-04	9.5E-04	4.8E-03	2.1E-03
Ovitrap *w*Mel prevalence	−	**2.14**	−	−	**1.56**	−	1.52	1.96	1.23
Lag 1 ovitrap *w*Mel prevalence	−	−	1.19	−	**1.54**	1.44	1.56	−	1.61
Month	−	−	−	0.022	−	−0.15	−0.033	0.18	0.19
Ovitrap *w*Mel prevalence*month interaction	N	**N**	N	N	**N**	N	N	Y	Y
Random effects									
Cluster (variance; SD)	1.39 (1.18)	**0.24 (0.49)**	0.77 (0.88)	1.41 (1.19)	**0.29 (0.54)**	0.57 (0.76)	0.66 (0.82)	0.24 (0.48)	0.27 (0.52)
Delta BIC	+28.79	**+2.52**	+28.86	+31.92	**0**	+31.37	+2.97	+7.98	+5.06

BG-traps = BG-Sentinel traps (Biogents AG; Regensburg, Germany); BIC = Bayesian Information Criterion; N = no; ovitrap = oviposition trap; Y = yes.

The relationship between the ovitrap and BG-trap data was modeled using a negative binomial regression approach. The cluster was included as a random effect in all models, as well as an offset using the log of the total number of *Aedes aegypti* caught using that BG-trap. The BIC is presented as 0 for the best-fitting model and as the difference in BIC for the other models.

The bolding signifies the best fitting models which were used to analyze ovitrap prediction.

Utilizing the 60% WMP introgression threshold, the overall sensitivity of Model 5’s introgression classification was found to be 73%, with a specificity of 89%, across clusters and time points, and Model 2’s sensitivity was 63%, with a specificity of 67% (Supplemental Table 8). Model 5 correctly classified 80% of the estimates but misclassified four estimates (20%), with three overestimated and one underestimated. Model 2 correctly classified 65% of the estimates but misclassified seven estimates (35%), with three overestimated and four underestimated.

## DISCUSSION

Interventions with *w*Mel *Wolbachia* are poised to play a significant role in the future of arboviral disease control, given the mounting evidence of their ability to reduce arboviral infection disease risk. Resource constraints may hamper efforts to scale up this intervention. In the present study, the authors aimed to determine the predictive precision of a low-resource monitoring strategy, ovitrap surveillance, in a post-release phase of a *w*Mel program compared with the more resource-intensive BG-traps. Using the sampling strategy and cluster demarcations of the EVITA dengue randomized controlled trial and municipal arboviral surveillance in Belo Horizonte, Brazil, the introgression estimation capabilities of ovitraps and BG-traps were compared. Data from ovitraps are highly correlated with those from BG-traps and serve as a good proxy for *w*Mel-positive introgression estimates from BG-traps. However, given the limited number of months of evaluation, further work is needed to assess the generalizability of ovitrap results and their accuracy relative to BG-traps.

Cluster-level *w*Mel introgression estimates were highly correlated between ovitraps and BG-traps over time. Traps were sampled in March, April, and May. This fall period corresponds to a transition from high to low *Aedes* mosquito density in Belo Horizonte, providing the opportunity to compare the traps’ introgression monitoring through a range of mosquito population densities. Although the overall sensitivity and specificity of ovitrap introgression classification were moderate (67% and 61%, respectively), half of the misclassifications occurred in March. Eggs from March ovitrap paddles were stored until testing in early June; therefore, the misclassification may be due in part to the proven lowered quiescent viability of *w*Mel-infected eggs. Allman et al. (2023) found significant reductions in *w*Mel and wildtype egg hatch rates in eggs stored for longer than 4 weeks.^30^ Therefore, to avoid this misclassification in the future, eggs should not be stored for longer than 4 weeks before *w*Mel detection is performed.

In the present regression modeling analysis, accounting for the previous month’s ovitrap *w*Mel-positive count, the prevalence of *w*Mel in ovitraps in both the current month and the previous month, and information on *Ae. aegypti* abundance, ovitrap estimates were highly associated with the current month’s count of *w*Mel-positive in BG-traps. In the best-fitting predictive model (Model 5), this association was leveraged to estimate *w*Mel counts and prevalence estimates with greater precision than just using ovitrap introgression estimates alone. Given that the models do not include a monthly temporal term, their results are likely generalizable to other time periods if a measure of *Ae. aegypti* abundance is known (i.e., the offset included in the study models). With low error and relatively high sensitivity and specificity, the best-fitting predictive model provides a framework for future use of ovitrap monitoring to determine *w*Mel introgression.

In the present study, the comparative predictive precision of ovitraps and BG-traps is investigated for the monitoring of *w*Mel introgression in a post-release monitoring phase of *w*Mel releases using existing *Aedes* surveillance networks. Although ovitraps are inexpensive, sensitive, and scalable tools for detecting *Aedes* population dynamics, they are inherently limited in their ability to represent the host-seeking adult mosquito population because they rely on passive, oviposition-based sampling.^8^^,^^32^ Their output can be influenced by factors such as egg viability, larval competition, and species composition, and they may not track adult abundance accurately under high-density or varied ecological conditions.^33^ In contrast, BG-traps are active systems designed to selectively capture host-seeking adult females, thereby offering a more direct measure of circulating vector populations.^33^^,^^34^ However, there is the possibility that BG-traps could overestimate *w*Mel prevalence because of false positives in BG-trap collections of females; it has been observed in the laboratory that *w*Mel-uninfected females that have mated with *w*Mel-infected males have low levels of *w*Mel *Wolbachia*.^35^ Comparative field studies across diverse settings—including Brazil, Germany, Italy, and South Africa—consistently reveal the superior efficacy of BG-traps in capturing adult mosquitoes across species and habitats, although ovitraps reliably reflect seasonal trends and often avoid zero capture events, even at low densities.^8^^,^^32^^,^^36^ Although BG-traps may also collect males looking for females to mate (the sex of the *Ae. aegypti* captures was not determined in the present study), measuring *w*Mel prevalence in both sexes is important to ensure population replacement. *Wolbachia* prevalence in males is crucial for introgression because of the reliance on the frequency-dependent fitness advantages of *Wolbachia* infection, which confer cytoplasmic incompatibility during mating between a *Wolbachia*-infected male and a wild-type female. Previous work conducted in Rio de Janeiro revealed the potential utility of ovitraps for *w*Mel monitoring, showing strong concordance with BG-trap estimates in semi-field and field settings and low sampling error under controlled conditions.^7^ The authors of the work found a sampling error of less than 0.1% using ovitraps in their semi-field system and found similar predictive abilities between the ovitraps and BG-traps in the Urca neighborhood. The authors of the present study build upon this foundation by evaluating both ovitrap and BG-trap performance under operational, post-release conditions across a wider geographic region (480–2,400 hectares [only a range of cluster sizes is known because of blinding] compared with the 8.6 hectares studied in the Rio study) and during a different seasonal period. Embedded within the structured design of the EVITA dengue cluster-randomized controlled trial, a predictive modeling framework is used to compare trap types in terms of their utility for estimating local *w*Mel prevalence. These findings contribute to the growing evidence base supporting the integration of ovitraps into large-scale *Wolbachia* monitoring programs while acknowledging their context-dependent constraints.

Although the present study has numerous strengths, it also has certain inherent limitations. Given the ongoing nature of the EVITA dengue trial, no spatial information was available for the traps. Although this maintained the blinding of the investigators, it limited the resolution at which associations could be made at the cluster level. However, the size of the clusters is representative of the scale the WMP uses to monitor introgression and inform the need for continued releases in other Brazilian cities. Thus, understanding the correlation at this scale is informative for the decision-making process. Further exploration is needed to determine the spatial correlation between BG-traps and ovitraps in close proximity and the resolution of the prediction that the trap network produces. However, by investigating the within-cluster variance of both trap types, the authors found consistently low variance for both, which suggests relatively low trap-to-trap heterogeneity within clusters. In tandem with the high autocorrelation of BG-trap estimates found in the sensitivity analysis, it is unlikely that trap positioning drives large trap-to-trap differences in prevalence estimates. In addition, after bootstrap resampling and running correlation tests 10,000 times to replicate the statistical uncertainty associated with trap placement, the correlation estimate held. Both ovitrap-derived and model-predicted prevalence estimates exhibited misclassification using a 60% introgression threshold, which may pose operational challenges because the WMP gauges when to stop releases in the consolidation phase based on this threshold. However, the majority of estimates were correctly classified, and the authors hypothesize that the misclassification may have been due in part to the storage time of the eggs. In the future, storage of ovitrap paddles before rearing and testing should be limited. In the release and consolidation phases of release programs, *w*Mel introgression is likely to be <40%, a threshold for which the models exhibited little misclassification. Larvae hatched from ovitrap collections could not be morphologically speciated because of limited personnel and access to microscopes for identification. To account for this, the sample size used for testing ovitrap collections was increased. Additionally, although ovitraps have been found to be less resource-intensive by the WMP and are used in municipal passive surveillance programs, a full cost-effectiveness study was outside the scope of the present study. Future analyses are needed to fully understand the potential resource tradeoffs between the trap types. Finally, the authors were unable to perform out-of-sample validation of the models because of the limited temporal extent of the data. Future ovitrap data collection will enable external validation of the model.

## CONCLUSION

Although *Wolbachia*-based interventions have shown promise in reducing vector-borne disease transmission, scaling these interventions for broad implementation in resource-limited settings remains challenging. A crucial aspect of this challenge is the need for efficient monitoring of *Wolbachia* introgression after the release of *w*Mel-infected *Ae. aegypti* mosquitoes. Effective surveillance of introgression and wild-type populations is essential for adjusting release strategies to ensure successful establishment. The present study reveals a strong correlation between *w*Mel prevalence estimates derived from ovitraps and those derived from BG-traps, supporting ovitraps as a cost-effective proxy for assessing *Wolbachia* introgression. Furthermore, the findings suggest that predictive models incorporating previous introgression data and mosquito abundance can further enhance the accuracy of ovitrap-based estimates. This evidence underscores the utility of ovitraps in ongoing monitoring and long-term assessment of W*olbachia* interventions, providing a scalable, low-resource tool that could facilitate widespread roll-out in low-income regions.

## Supplemental Materials

10.4269/ajtmh.26-0050 Supplemental Materials
