# Supplementary material for: Comparison of Oviposition and Adult Trapping to Monitor wMel Introgression for Wolbachia-Based Vector Control
Source: Am J Trop Med Hyg. 2026 Jul 21;115(3):483–92. doi: 10.4269/ajtmh.26-0050 (PMC13539766; doi:10.4269/ajtmh.26-0050)
Supplement: Supplementary file 1 [file tpmd260050.SD1.pdf]

Supplemental Materials

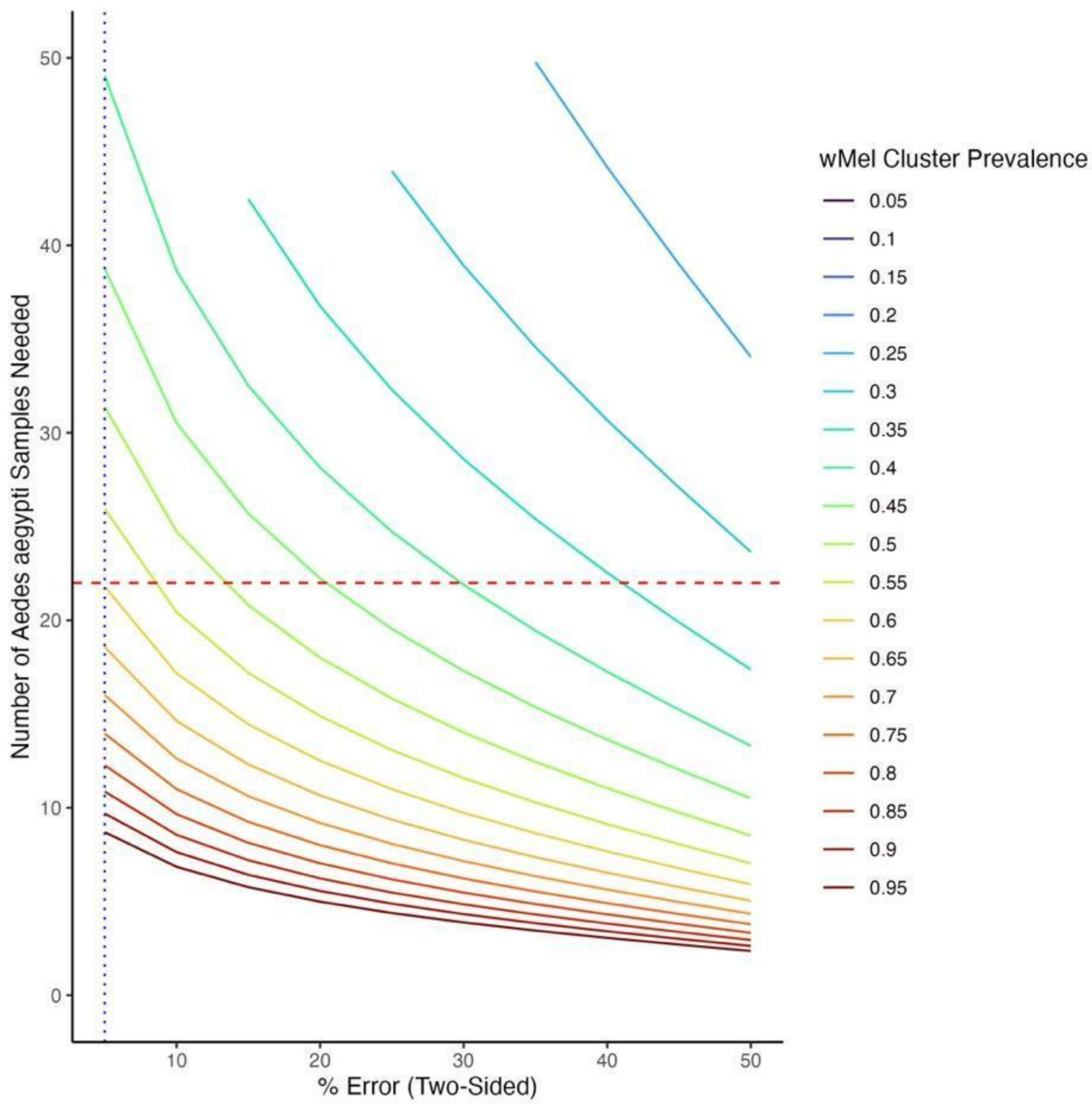

Figure S1. Significance analysis for oviposition trap larvae sample size. The number of *Aedes aegypti* larvae samples needed at different levels of *w*Mel prevalence and significance levels are indicated by the colored lines, calculated using a two-sided power calculation for proportion test (one sample) from the 'pwr' package in R. The red horizontal line indicates the chosen sample size of 22 *Ae. aegypti* larvae. The blue dashed vertical line indicates 5% two-sided error.

Table S1. BG-Sentinel trap sample collection and testing results. Data is presented at the cluster level and combined across the three time points (March, April, May). Adult mosquitoes were trapped, morphologically-identified to the species level, and tested by WMP.

Cluster	No. of Traps[1]	Total No. of Adults Caught[2]	No. of *Ae. albopictus* Caught[3]	No. of *Ae. aegypti* Tested[4]	No. of *w*Mel-positive *Ae. aegypti* tested[5]
**A**	21 (8.9)	99 (6.3)	2 (2)	83 (83.8)	67 (80.7)
**B**	24 (10)	144 (9)	0 (0)	140 (97.2)	112 (80)
**C**	11 (4.6)	55 (3.5)	1 (1.8)	51 (92.7)	41 (80.4)
**D**	24 (10)	124 (7.9)	1 (0.8)	81 (65.3)	64 (79)
**E**	14 (5.8)	115 (7.3)	1 (0.9)	81 (70.4)	49 (60.5)
**F**	23 (9.6)	107 (6.8)	3 (2.8)	94 (87.9)	56 (59.6)
**G**	14 (5.8)	90 (5.7)	1 (1.1)	65 (72.2)	45 (69.2)
**H**	24 (10)	84 (5.3)	1 (1.2)	79 (94.1)	55 (69.6)
**I**	14 (5.8)	105 (6.6)	0 (0)	84 (80)	40 (47.6)
**J**	23 (9.6)	186 (11.8)	0 (0)	165 (88.7)	18 (10.9)
**K**	14 (5.9)	95 (6)	0 (0)	58 (61.1)	3 (5.2)
**L**	31 (14)	376 (23.8)	2 (0.5)	263 (70)	8 (3)

[1] % out of 237 total traps across clusters
[2] % out of 1,580 total adults caught across clusters
[3] % out of adults caught in that cluster
[4] % out of adults caught in that cluster
[5] % of *Ae. aegypti* tested in that cluster

Table S2. Oviposition trap sample collection and testing results. Data is presented at the cluster level and combined across the three time points (March, April, May). Eggs were trapped and collected by the Belo Horizonte Municipal Health Department. Eggs were hatched, reared, and tested by the authors. Eggs were counted by the Municipality in March and April, and by an author in May.

Cluster	No. of Traps[1]	Total No. of	No. of Larvae Hatched[3]	No. of Larvae Tested[4]	No. of *Ae. albopictus* identified[5]	No. of *Ae. aegypti* identified[6]	No. of *w*Mel-positive[7]

		Eggs Caught[2]					
**A**	9 (7.3)	695 (4.7)	320 (46)	229 (72)	18 (7.9)	210 (92)	199 (95)
**B**	10 (8.1)	1299 (8.6)	735 (57)	447 (61)	47 (10)	400 (90)	305 (76)
**C**	8 (6.5)	502 (3.4)	252 (50)	116 (46)	3 (2.6)	108 (93)	92 (85)
**D**	11 (8.9)	489 (3.3)	193 (40)	165 (86)	66 (40)	99 (60)	67 (68)
**E**	8 (6.5)	1073 (7.2)	493 (46)	190 (39)	43 (23)	147 (77)	139 (95)
**F**	14 (11)	3249 (21)	940 (29)	493 (52)	56 (11)	437 (89)	290 (66)
**G**	8 (6.5)	706 (4.7)	367 (52)	257 (70)	103 (40)	154 (60)	82 (53)
**H**	15 (12)	2338 (15)	979 (42)	610 (62)	81 (12)	529 (88)	278 (53)
**I**	10 (8.1)	420 (2.8)	145 (35)	134 (92)	8 (6)	125 (93)	67 (54)
**J**	10 (8.1)	1624 (11)	1225 (75)	557 (46)	97 (17)	460 (83)	23 (5)
**K**	5 (4)	802 (5.3)	219 (27)	190 (87)	5 (3)	185 (97)	0 (0)
**L**	16 (13)	2045 (13)	1051 (51)	377 (36)	82 (22)	295 (78)	18 (6.1)

[1] % out of 124 total traps across clusters
[2] % out of 15,242 total eggs caught across clusters
[3] % out of eggs caught in that cluster
[4] % out of larvae hatched in that cluster
[5] % of larvae tested in that cluster
[6] % of larvae tested in that cluster
[7] % of *Ae. aegypti* tested in that cluster

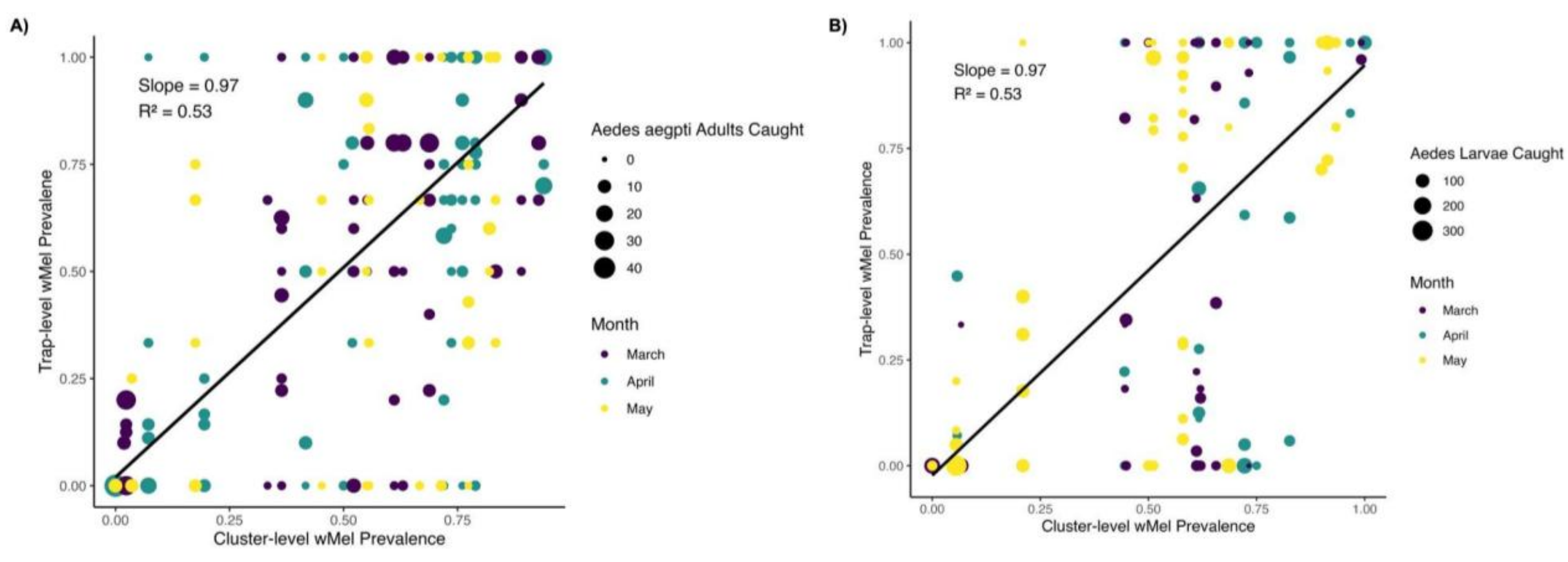

Figure S2. Cluster-level vs. trap-level *w*Mel prevalence. Distribution of cluster-level vs. trap-level *w*Mel prevalence estimates in BG-traps (A) and ovitraps (B). Points increase in size based on the total number of *Ae. aegypti* caught in each BG-trap and the total number of *Aedes* larvae hatched from eggs from each ovitrap. The color of the points indicate the month of collection.

The black line indicates the correlation between the cluster-level and trap-level estimates using inverse variance weighting using the number of total *Ae. aegypti*. The slope of the correlation line and the R^2 of the associated model are listed in the top left.

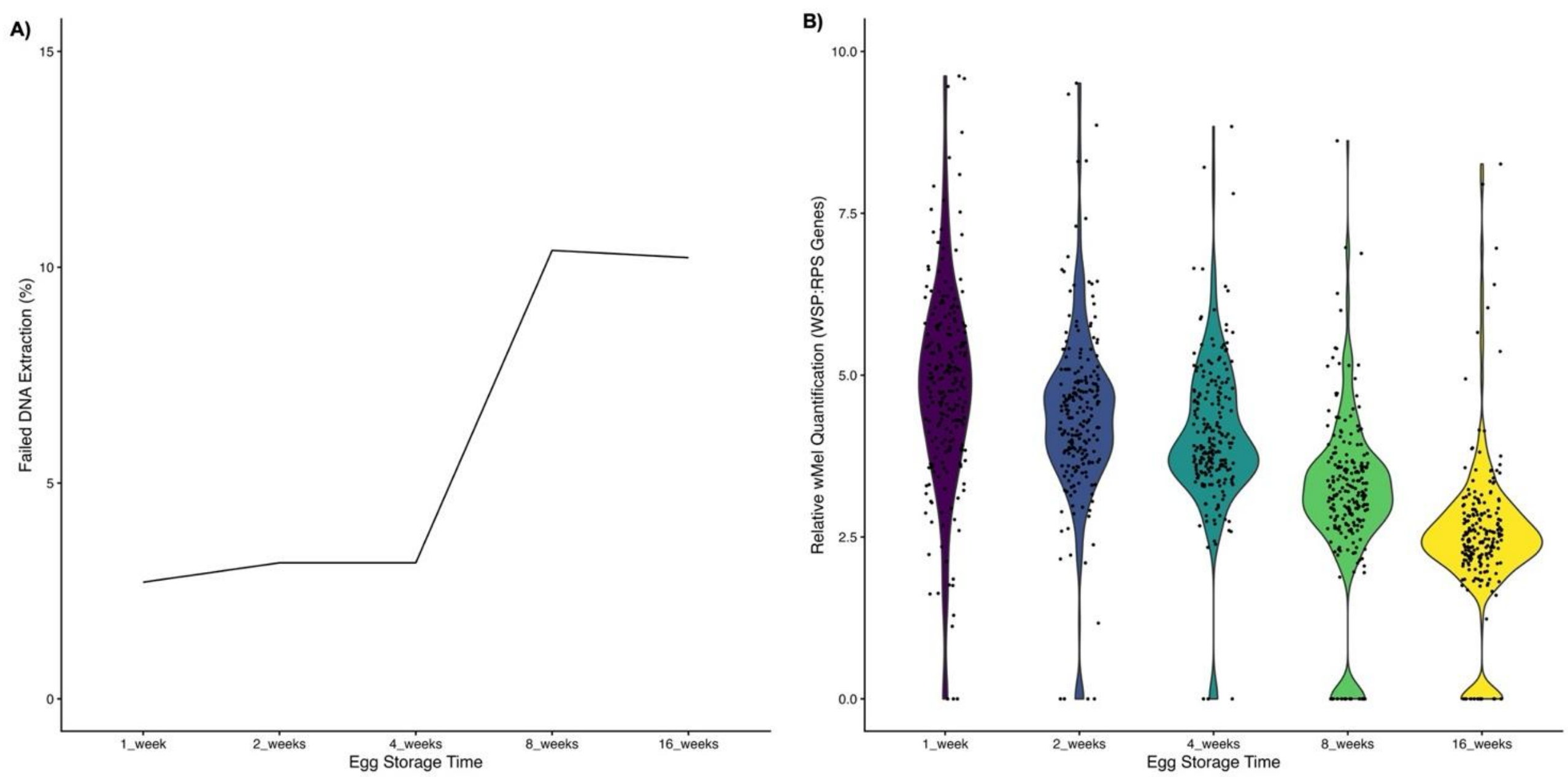

Figure S3. The effect of *w*Mel-positive *Ae. aegypti* egg storage time on *w*Mel DNA extraction failure rates and relative density. *w*Mel eggs from World Mosquito Program's *w*Mel-positive *Ae. aegypti* colonies were stored for 1, 2, 4, 6, 8, or 16 weeks. All eggs were stored in a controlled environment until testing, which followed WMP's standard *w*Mel detection qPCR protocol. A) We defined DNA extraction failure as the absence of detectable amplification in the qPCR assays. For each storage time point, we calculated the percentage of wMel eggs tested that showed no Ct value. B) We calculated the relative *w*Mel density (bacteria-to-host ratio) by quantifying the copy numbers for the *w*Mel bacterial gene (WSP) and the *Aedes*-specific housekeeping gene (RPS17) across storage time points. The data points represent

individual *w*Mel eggs and are jittered. The underlying distribution of the points is shown using a violin plot.

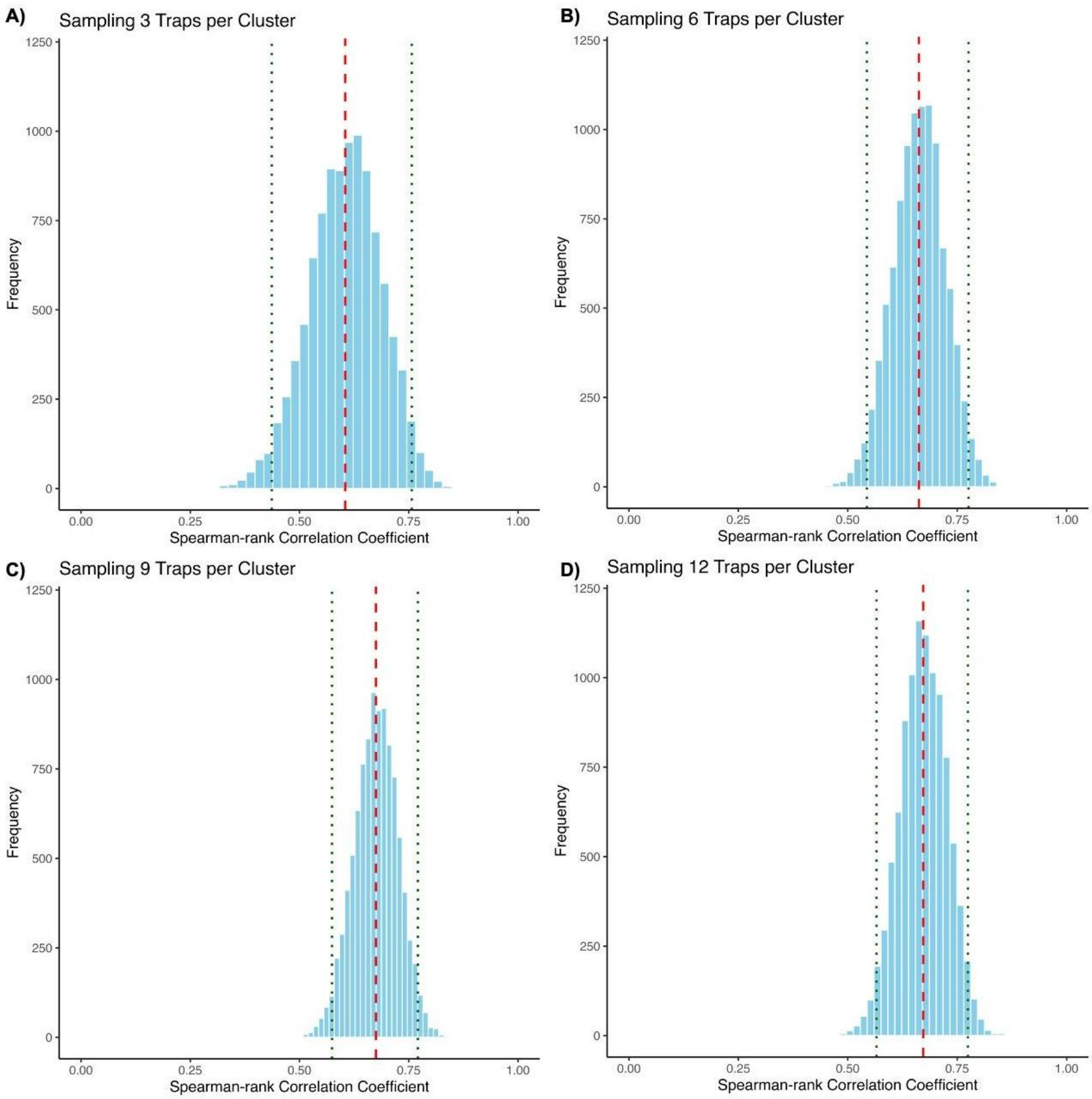

Figure S4. Distribution of Spearman-rank correlation coefficients of cluster-level estimates of wMel introgression derived from ovitraps with those derived from BG-sentinel traps from bootstrap resampling of traps within clusters to replicate spatial uncertainty. To quantify potential uncertainty in the spatial aggregation to cluster level, we bootstrap resampled various numbers of ovitraps and BG-traps from each cluster to include in generating cluster-level

metrics. We performed bootstrap resampling 10,000 times with replacement of introgression estimates and tested for correlation using a Spearman-rank correlation test. The red dashed line indicates the mean correlation coefficient estimate of A) 0.61 (95% CI 0.44-0.76) using 3 traps, B) 0.66 (95% CI 0.54-0.78) using 6 traps, C) 0.68 (95% CI 0.58-0.77) using 9 traps, and D) 0.67 (95% CI 0.57-0.78) using 12 traps. The dark green dotted lines indicate the 95% confidence interval.

Table S3. BG-Sentinel trap unadjusted prevalence estimates. Estimates are calculated by averaging trap-level proportions within each cluster at each time point. The 95% binomial confidence interval is included after the prevalence point estimate.

Cluster	March	April	May
**A**	0.93 (0.81-0.98)	0.74 (0.57-0.86)	0.71 (0.61-0.80)
**B**	0.89 (0.76-0.95)	0.76 (0.64-0.85)	0.77 (0.69-0.84)
**C**	0.83 (0.57-0.95)	0.79 (0.62-0.90)	0.83 (0.62-0.94)
**D**	0.63 (0.46-0.77)	0.94 (0.82-0.98)	0.45 (0.34-0.57)
**E**	0.69 (0.54-0.81)	0.72 (0.55-0.84)	0.83 (0.72-0.91)
**F**	0.52 (0.38-0.67)	0.79 (0.63-0.89)	0.56 (0.47-0.64)
**G**	0.61 (0.45-0.75)	0.50 (0.27-0.73)	0.82 (0.69-0.90)
**H**	0.55 (0.36-0.73)	0.52 (0.33-0.70)	0.55 (0.44-0.65)
**I**	0.36 (0.24-0.51)	0.42 (0.45-0.59)	0.67 (0.51-0.79)
**J**	0.02 (0.004-0.08)	0.19 (0.11-0.32)	0.18 (0.11-0.26)
**K**	0.33 (0.08-0.76)	0.00 (0.00-0.10)	0.03 (0.01-0.11)
**L**	0.02 (0.008-0.70)	0.07 (0.04-0.14)	0.00 (0.00-0.03)

Table S4. Oviposition trap unadjusted prevalence estimates. Estimates are calculated by averaging trap-level proportions within each cluster at each time point. The 95% binomial confidence interval is included after the prevalence point estimate. Estimates marked as "NA" refer to time point without any *Aedes aegypti* larvae samples collected.

Cluster	March	April	May
**A**	0.99 (0.91-1.00)	0.97 (0.91-0.99)	0.90 (0.82-0.95)
**B**	0.66 (0.57-0.73)	0.83 (0.77-0.87)	0.51 (0.42-0.60)

**C**	0.45 (0.31-0.59)	1.00 (0.95-1.00)	NA
**D**	0.50 (0.39-0.61)	NA	0.93 (0.86-0.97)
**E**	0.73 (0.53-0.87)	1.00 (0.94-1.00)	0.91 (0.84-0.95)
**F**	0.61 (0.52-0.70)	0.72 (0.66-0.78)	0.69 (0.60-0.76)
**G**	0.45 (0.34-0.56)	0.75 (0.66-0.82)	0.50 (0.39-0.61)
**H**	0.62 (0.52-0.71)	0.62 (0.55-0.68)	0.58 (0.52-0.63)
**I**	0.61 (0.46-0.73)	0.44 (0.35-0.55)	NA
**J**	0.00 (0.00-0.02)	0.00 (0.00-0.02)	0.21 (0.16-0.27)
**K**	0.00 (0.00-0.05)	0.00 (0.00-0.06)	0.00 (0.00-0.06)
**L**	0.07 (0.03-0.13)	0.06 (0.03-0.11)	0.06 (0.03-0.11)

Table S5. Ovitrap introgression threshold classification sensitivity and specificity. WMP uses 0.6 prevalence in an area as an introgression threshold to determine whether introgression will self-sustain or whether further releases are needed. The prevalence estimates from ovitraps were compared to those from BG-traps for all clusters and time points, and classification above or below the varying thresholds were used to construct 2x2 tables. Sensitivity and Specificity were calculated across clusters for each time point from the 2x2 tables.

	**March**	**April**	**May**
**60% Threshold**			
Sensitivity (%)	50 (3/6)	100 (5/5)	50 (2/4)
Specificity (%)	50 (3/6)	67 (4/6)	67 (4/6)
**55% Threshold**			
Sensitivity (%)	57 (4/7)	100 (5/5)	67 (4/6)
Specificity (%)	60 (3/5	67 (4/6)	75 (3/4)
**50% Threshold**			
Sensitivity (%)	75 (6/8)	100 (7/7)	100 (6/6)
Specificity (%)	75 (3/4)	100 (4/4)	75 (3/4)
**45% Threshold**			
Sensitivity (%)	100 (8/8)	100 (7/7)	100 (7/7)
Specificity (%)	75 (3/4)	75 (3/4)	100 (3/3)
**40 % Threshold**			
Sensitivity (%)	100 (8/8)	100 (8/8)	100 (7/7)
Specificity (%)	75 (3/4)	100 (3/3)	100 (3/3)
**35% Threshold**			
Sensitivity (%)	100 (9/9)	100 (8/8)	100 (7/7)
Specificity (%)	100 (3/3)	100 (3/3)	100 (3/3)

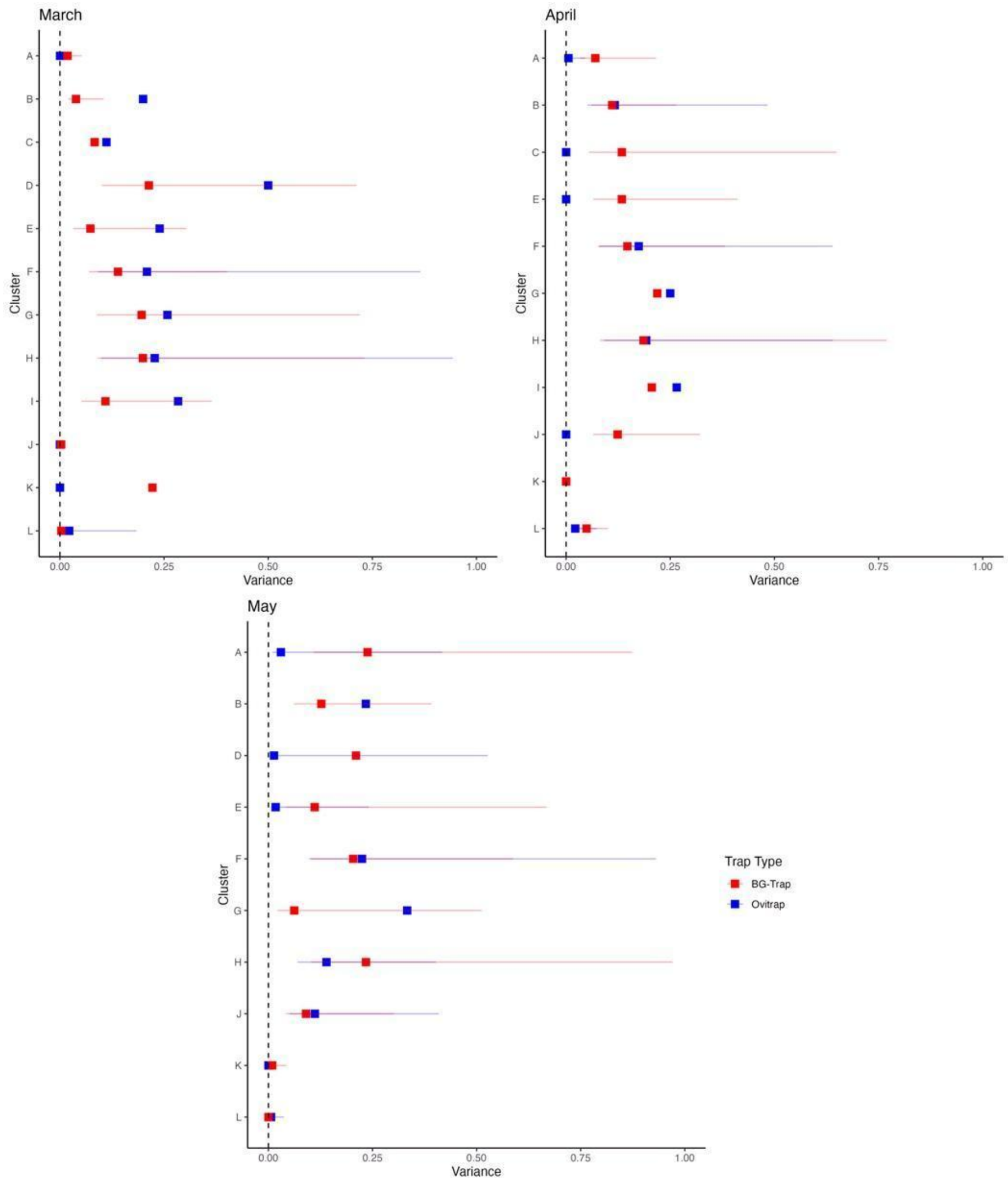

Figure S5. Within-Cluster *w*Mel Prevalence Variation. The within-cluster variance of trap-level prevalence estimates was calculated and averaged by cluster and time point, along with the chi-square 95% confidence interval. The forest plots above show the distribution of the cluster-level variance by month. The plot was created using the *ggplot2* package in R. The variance across clusters for BG-traps was 0.11 in March, 0.13 in April, and 0.13 in May. For ovitraps it was 0.17 in March, 0.09 in April, and 0.11 in May.

Table S6. Temporal correlation to inform variable selection for regression model. Correlation estimates were calculated using a spearman-rank correlation test on each cluster's *w*Mel count by time point. Given that only three time points were available for each cluster, there is not enough data for a significant p-value. Similarly, Pearson correlation tests were unable to run with the limited data.

Cluster	BG vs. Ovi Count Correlation	*P*-Value	BG vs. Lag 1 Month Ovi Correlation	*P*-Value	Ahead 1 Month BG vs. Ovi Correlation	*P*-Value
**A**	-0.50	1.00	1.00	1.00	1.00	1.00
**B**	-0.50	1.00	1.00	1.00	1.00	1.00
**C**	1.00	1.00	1.00	1.00	1.00	1.00
**D**	1.00	1.00	NA	NA	NA	NA
**E**	0.50	1.00	1.00	1.00	-1.00	1.00
**F**	0.50	1.00	1.00	1.00	1.00	1.00
**G**	-1.00	0.34	1.00	1.00	1.00	1.00
**H**	0.50	1.00	1.00	1.00	1.00	1.00
**I**	-1.00	1.00	1.00	1.00	1.00	1.00
**J**	0.87	0.34	NA	NA	NA	NA
**K**	NA	NA	NA	NA	NA	NA
**L**	0.00	1.00	-1.00	1.00	1.00	1.00

Table S7. Model predicted *w*Mel counts error. Error was calculated by subtracting the true count from the model-predicted count and dividing by the true count. Negative error indicates an underestimation by the model, whereas positive error indicates an overestimation. NA resulted from a lack of ovitrap data at the time point or the previous time point. If 0 *w*Mel-positive were caught by BG-traps in that cluster at the time point, that estimate was excluded.

Cluster	Model 5			Model 2		
	**April**	**May**	**Overall**	**April**	**May**	**Overall**
**A**	-0.04	0.50	0.23	-0.13	0.63	0.25
**B**	0.02	-0.08	-0.03	0.09	-0.21	-0.06
**C**	-0.05	NA	-0.05	-0.01	NA	-0.01

**D**	NA	NA	NA	NA	NA	NA
**E**	0.06	0.23	0.15	0.12	-0.12	-0.001
**F**	-0.16	0.17	0.006	-0.25	0.30	0.03
**G**	-0.02	-0.09	-0.05	0.22	-0.30	-0.04
**H**	0.17	-0.16	0.004	0.002	-0.09	-0.04
**I**	-0.06	NA	-0.06	-0.12	NA	-0.12
**J**	-0.16	-0.15	-0.15	-0.16	-0.04	-0.10
**K**	NA	-0.29	-0.29	NA	-0.10	-0.10
**L**	0.07	NA	0.07	0.22	NA	0.22
**Across Clusters**	-0.02	0.02	0.0004	-0.002	0.01	0.004

Table S8. Model predicted introgression success classification sensitivity and specificity. WMP uses 0.6 prevalence in an area as an introgression threshold to determine whether introgression will self-sustain or whether further releases are needed. The prevalence predicted by the models were compared to those from BG-traps across clusters and time points, and classification above or below the 0.6 threshold were used to construct 2x2 tables. Sensitivity and Specificity were calculated across clusters for each time point from the 2x2 tables.

	**Model 5**		**Model 2**	
	**April**	**May**	**April**	**May**
**Sensitivity (%)**	80 (4/5)	80 (4/5)	80 (4/5)	25 (1/4)
**Specificity (%)**	84 (5/6)	100 (3/3)	84 (5/6)	50(3/6)

Table S9. Autocorrelation tests to determine spatial correlation of BG-traps. Correlation estimates were calculated using a spearman-rank correlation test on each cluster's *w*Mel count by time point. Given that only three time points were available for each cluster, there is not enough data for a significant p-value.

Cluster	BG vs BG Correlation	*P*-Value	BG vs. Lag 1 Month BG Correlation	*P*-Value	BG vs. Ahead 1 Month BG Correlation	*P*-Value
**A**	1.00	0.34	-1.00	1.00	1.00	1.00
**B**	1.00	0.34	1.00	1.00	1.00	1.00
**C**	1.00	0.34	1.00	1.00	-1.00	1.00
**D**	1.00	0.34	1.00	1.00	1.00	1.00
**E**	1.00	0.34	-1.00	1.00	NA	NA
**F**	1.00	0.34	1.00	1.00	-1.00	1.00
**G**	1.00	0.34	-1.00	1.00	-1.00	1.00
**H**	1.00	0.34	-1.00	1.00	1.00	1.00
**I**	1.00	0.34	-1.00	1.00	-1.00	1.00
**J**	1.00	0.34	1.00	1.00	1.00	1.00
**K**	1.00	0.34	-1.00	1.00	-1.00	1.00
**L**	1.00	0.00	NA	NA	-1.00	1.00
